# On the simple calculation of walking efficiency without kinematic information for its convenient use

**DOI:** 10.1186/s40101-019-0211-4

**Published:** 2019-12-30

**Authors:** Daijiro Abe, Yoshiyuki Fukuoka, Masahiro Horiuchi

**Affiliations:** 1 Biodynamics Laboratory, Center for Health and Sports Science, Kyushu Sangyo University, 2-3-1 Matsukadai, Higashi-ku, Fukuoka City, Fukuoka, 813-8503 Japan; 2 Faculty of Health and Sports Science, Doshisha University, Kyotanabe City, Kyoto Japan; 3 Division of Human Environmental Science, Mount Fuji Research Institute, Fujiyoshida City, Yamanashi Japan

**Keywords:** Bipedalism, Locomotion, Gait, Model analysis

## Abstract

**Background:**

Since walking is a daily activity not to require the maximal effort in healthy populations, a very few universal bio-parameters and/or methods have been defined to evaluate individual walking characteristics in those populations. A concept of “economy” is a potential candidate; however, walking economy highly depends on speed, so direct comparisons of economy values are difficult between studies. We investigated whether the vertical component of net walking “efficiency” (Eff_vert_; %) is constant across speed. In that case, direct comparisons of Eff_vert_ will be possible between studies or individuals at any voluntary speed.

**Methods:**

Thirty young male participants walked at eight speeds on the level or ± 5% gradients, providing vertical speeds (*v*_vert_). Differences in energy expenditure between level and uphill or downhill gradients (ΔEE) were calculated. The metabolic rate for vertical component (MR_vert_) was calculated by multiplying ΔEE with body mass (BM). The mechanical power output for vertical component (P_mech_) was calculated by multiplying BM, gravitational acceleration, and *v*_vert_. Eff_vert_ was obtained from the ratio of P_mech_ to MR_vert_ at each *v*_vert_. Delta efficiency (Delta-E; %) was also calculated from the inverse slope of the regression line representing the relationship of P_mech_ to MR_vert_.

**Results:**

Upward Eff_vert_ was nearly constant at around 35% and downward Eff_vert_ ranged widely (49–80%). No significant differences were observed between upward Delta-E (35.5 ± 8.8%) and Eff_vert_ at any speeds, but not between downward Delta-E (44.9 ± 12.8%) and Eff_vert_.

**Conclusions:**

Upward ΔEE could be proportional to *v*_vert_. Upward, but not downward, Eff_vert_ should be useful not only for healthy populations but also for clinical patients to evaluate individual gait characteristics, because it requires only two metabolic measurements on the level and uphill gradients without kinematic information at any voluntary speed.

**Trial registration:**

UMIN000017690 (R000020501; registered May 26th, 2015, before the first trial) and UMIN000031456 (R000035911; registered Feb. 23rd, 2018, before the first trial).

## Background

Walking and running are two of the major gait patterns in the erect bipedal locomotion. Previous literatures have evaluated individual running capacities using already established physiological parameters. However, except for race walking, walking is a daily activity not to require individual maximal effort, so relatively fewer biomechanical parameters or methods have been developed to evaluate individual walking characteristics in healthy populations and clinical patients [1]. A concept of “efficiency” is a potential and universal candidate; however, the evaluation of walking efficiency takes considerable technical effort because of the need to process kinematic information using expensive devices and specialized software [1, 2]. Nevertheless, it still involves various uncertainties in the quantification of the mechanical work done by multiple body segments (internal work), as well as the “negative” work [2]. Thus, literatures often use the concept of “economy,” the energy cost of transport per unit distance (CoT; J·kg^−1^·km^−1^), because this only requires metabolic information [3]. However, walking economy is highly dependent on speed, with a U-shaped relationship between CoT values and walking speed [4–7]. Thus, as far as we know, the economical speed could be the only index for evaluating walking ability in each individual [4–11]. However, it is difficult to measure the entire CoT-speed curve for people with poor physical fitness, such as patients after surgery [8], elderly populations [7, 9, 10], and pregnant women [11], for safety considerations. This may limit the number of metabolic measurements only at slower speeds. Differences in walking speed also make it difficult to directly compare economy values between studies.

For a given walking speed, whole-body energy expenditure (EE; J·kg^−1^·s^−1^) differs between the level and uphill/downhill gradients (ΔEE; J·kg^−1^·s^−1^); thus, the vertical component of metabolic rate (MR_vert_; watt) can be obtained by multiplying ΔEE with body mass (BM; kg). The vertical component of mechanical power output (*P*_mech_; watt) can be calculated by multiplying BM, gravitational acceleration (*g*; m·s^−2^), and the vertical component of walking speed (*v*_vert_; m·s^-1^) [12–16]. The ratio of the *P*_mech_ to MR_vert_ gives the vertical component of net walking efficiency (Eff_vert_; %). Here, Eff_vert_ at each speed will be possible to calculate without kinematic information.

The net “horizontal” walking efficiency calculated from both metabolic and kinematic information has varied widely between literatures (25–40%) [17–20]. It has also been reported to have an inverse U-shaped profile as a function of horizontal walking speed [18, 20]. In contrast, Eff_vert_ as a function of speed has been estimated to be constant for each individual during uphill running [21] and climbing ergometer exercise [22]. This is because the EE required to lift the body was proportional to the gradient when walking at a given speed [23]. If Eff_vert_ is constant across speeds, then an assessment of Eff_vert_ requires only two metabolic measurements while walking at any voluntary speed along the level and a gentle uphill gradient. Indeed, several previous studies have used Eff_vert_ to evaluate effects of maximal strength training on gait characteristics in elderly cardiorespiratory patients [13, 14], schizophrenia patients with gait disturbance [12], and healthy elderly populations [16]. The metabolic measurement in those studies was always conducted at a particular speed (1.0 m·s^−1^); however, only one study measured healthy young populations [16], suggesting that we merely know about a potential usage of Eff_vert_ for other populations, particularly at faster walking speeds.

Delta efficiency (Delta-E; %), defined as the inverse slope of the regression line representing the relationship of mechanical power output to energy consumption rate, might also be useful for evaluating walking efficiency “without” kinematic information. Although Delta-E has been used for running in previous studies (e.g., [21]), it still requires several sets of metabolic measurements. There has been limited information for evaluating both Eff_vert_ and Delta-E for walking [24, 25]. For an individual, there is only one Delta-E value for each gradient, so it is impossible to evaluate whether Eff_vert_ is dependent on *v*_vert_. It is interesting to note that downhill Eff_vert_ gradually decreases as a function of walking speed [25]. This could be due to changes in the recovery rate of pendular energy transduction between kinetic energy and gravitational potential energy [2, 25, 26] and greater eccentric muscle contractions during downhill walking, being associated with the utilization of stored elastic energy [19, 20, 25]. Contrary to downhill walking, uphill walking makes us more exhausted compared to level walking, because more “positive” work in the exercising muscles is necessary during uphill walking than level or downhill walking [25]. We hypothesized that upward, but not downward, Eff_vert_ values would be constant across various *v*_vert_. To test this hypothesis and to expand a potential procedure of previous studies [12–16, 21, 23, 24], the aim of this study was to obtain Eff_vert_ and Delta-E values for a range of *v*_vert_ in healthy young participants without kinematic information.

## Methods

### Participants

**Fig. 1:**
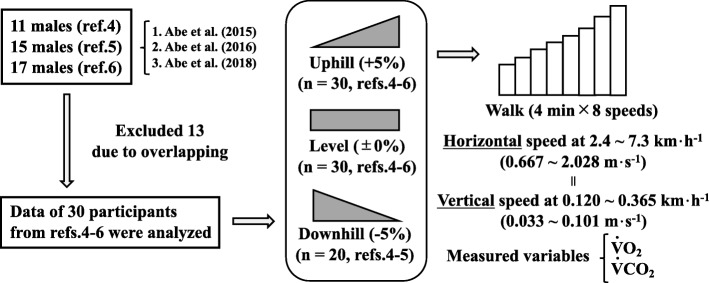
Number of participants on each gradient and measured conditions were schematically illustrated at eight walking speeds

As shown in Fig. 1, this study used additional data from already published investigations [4–6] from an entirely different perspective. Since several participants were overlapped among those investigations, the data from the first measurement in those overlapped participants were used in this study. Thirty males were involved in the level and uphill conditions with a mean age of 19.9 ± 1.0 years, stature of 170.5 ± 5.7 cm, and body mass of 59.8 ± 6.8 kg, respectively (mean ± standard deviation [SD]). Downhill gradient (− 5%) was not tested in one of the previous studies [6], so 20 of the 30 males (20.0 ± 1.1 years, 170.0 ± 6.3 cm, and 59.8 ± 5.5 kg) were involved in a downhill condition. An ethical committee of Kyushu Sangyo University approved all procedures of this study (H240324, H27-0002, and H28-0001).

### Study procedures

Exercise protocols were also described in Fig. 1. All participants continuously walked on a motor-driven treadmill (LABORDO LXE1200, Senoh, Japan) at eight horizontal walking speeds from 0.667 to 2.028 m s^−1^ on the level (± 0%) and uphill (+ 5% = + 2.862°) gradients. These horizontal speeds and gradients provided *v*_vert_ from 0.033 to 0.101 m s^−1^ calculated from a following equation [13–16].

1 $$ {v}_{\mathrm{vert}}=\mathrm{each}\ \mathrm{walking}\ \mathrm{speed}\cdotp \sin\ \left(2.862{}^{\circ}\right) $$

### Measurements and analysis

Oxygen uptake (VO_2_; mL·kg^–1^·min^–1^) and carbon dioxide output (VCO_2_; mL·kg^–1^·min^–1^) were continuously measured for 4 min at each speed with a breath-by-breath system (Additional file 1: Figure S1) (AE-310S, Minato Ltd, Japan). The average of VO_2_ and VCO_2_ for the final 2 min at each speed was used to calculate the energy expenditure (EE; J·kg^−1^·s^−1^) as follows [27].

2 $$ \mathrm{EE}=\frac{4.186\cdotp \left(3.869\cdotp V{O}_2+1.195\cdotp VC{O}_2\right)}{60} $$

ΔEE (J·kg^–1^·s^–1^) at each *v*_vert_ was multiplied by BM to obtain the MR_vert_:

3 $$ {\mathrm{MR}}_{\mathrm{vert}}=\varDelta \mathrm{EE}\cdotp \mathrm{BM} $$

*P*_mech_ was obtained by multiplying the BM, *g*, and *v*_vert_ [12–16, 21]:

4 $$ {P}_{\mathrm{mech}}=\mathrm{BM}\cdotp g\cdotp {v}_{\mathrm{vert}} $$

Eff_vert_ is given by the following equation. Because ΔEE was obtained by a subtraction between different gradients, the calculated Eff_vert_ was the net value.

5 $$ {\mathrm{Eff}}_{\mathrm{vert}}=\frac{P_{mech}}{M{R}_{vert}}\cdotp 100 $$

We also calculated the relationship between *P*_mech_ and MR_vert_ across eight measured *v*_vert_ for each participant on both gradients as follows:

6 $$ {\mathrm{MR}}_{\mathrm{vert}}=a\cdotp {\mathrm{P}}_{\mathrm{mech}}+b $$

where *a* and *b* are constants. Delta-E is the inverse slope of the regression line given in Eq. 6 [23, 24], so it can be calculated as follows:

7 $$ \mathrm{Delta}-\mathrm{E}=\frac{1}{\alpha}\cdotp 100 $$

Equation 7 means that each individual has only one Delta-E value for each gradient.

### Statistical analysis

The efficiency values were compared with one-way repeated measures ANOVA within participants on each gradient. When a significant *F* value was obtained, it was examined by Bonferroni/Dunn’s post hoc test. Statistical significance was accepted at *p* < 0.05.

## Results

**Fig. 2:**
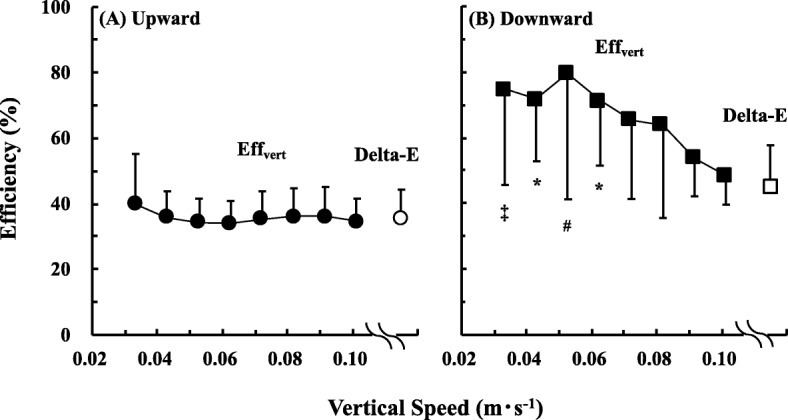
Upward (**a**) and downward (**b**) Eff_vert_, respectively. ^‡^ indicates *p* < 0.05 against 0.092, 0.101 m s^−1^ and Delta-E. * indicates *p* < 0.05 against Delta-E. ^#^ indicates *p* < 0.05 against 0.092, 0.101 m s^−1^, and Delta-E. Data are mean ± standard deviation (SD)

**Fig. 3:**
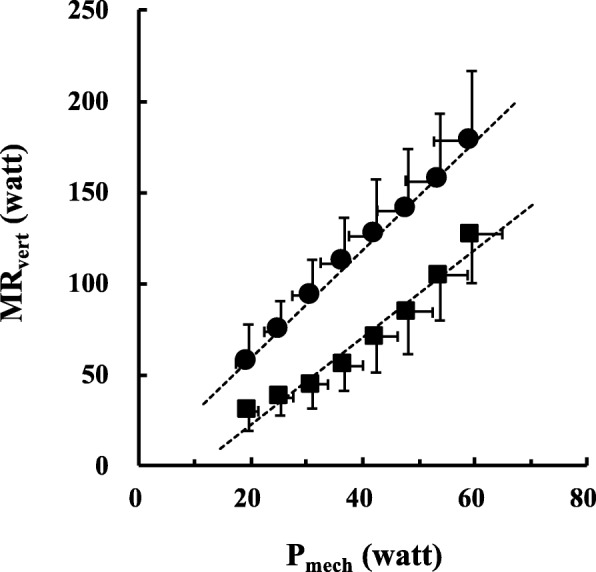
Upward (circles) and downward (squares) delta efficiency (Delta-E) was calculated from the inverse of the relationships of *P*_mech_ to MR_vert_ across eight measured vertical speed (*v*_vert_). MR_vert_ = 2.968·*P*_mech_ – 0.573 (*r* = 0.999) for upward Delta-E, and MR_vert_ = 2.399·*P*_mech_ – 25.665 (*r* = 0.981) for downward Delta-E, respectively. Data are mean ± SD

Upward Eff_vert_ values (range, 34.1–39.9%) did not differ significantly across measured *v*_vert_ (*F* = 1.045, *p* = 0.403; Fig. 2a and Additional file 2: Table S1). Downward Eff_vert_ values (range, 48.5–79.6%) were significantly higher at slower *v*_vert_ than at faster *v*_vert_ (*F* = 5.116, *p* < 0.001; Fig. 2b and Additional file 2: Table S1). Upward and downward Delta-E values were 35.5 ± 8.8% and 44.9 ± 12.8%, respectively (Fig. 3). There were significant differences between downward Delta-E and Eff_vert_ (Fig. 2b), but not between upward Delta-E and Eff_vert_ (Fig. 2a).

## Discussion

A striking finding of our study was that upward Eff_vert_ values did not vary significantly across the range of measured *v*_vert_ (Fig. 2a). However, downward Eff_vert_ values were significantly higher at slower *v*_vert_ than at faster *v*_vert_ (Fig. 2b). These results fully supported our hypothesis. The results indicated that ΔEE for the upward direction was proportional to *v*_vert_. That is, ΔEE for the upward direction can directly be explained by the amount of active muscles generating the accelerating force. This interpretation is consistent with those of some previous studies [18, 21, 22]. Because Eff_vert_ is a “dimensionless” value (%), it can directly be compared with the results of other studies, even if measured speed and/or gradient were different. On the basis of these interpretations, participant’s attribution could not be a limitation. For example, upward Eff_vert_ was significantly improved in schizophrenia patients with gait disturbance by 19.7% after 8-week maximal strength training [12]. This percent increase in Eff_vert_ is not surprising, because more considerable improvement was observed in healthy elderly populations [16] and cardiorespiratory patients [13, 15] after that training. A possible mechanism has been reported to be a high level of stress on all motor units including muscle activation [15]. Of note, these upward Eff_vert_ values in the previous studies were evaluated only at 1.00 m s^−1^ [12–16].

As far as we know, economical speed (ES) has been the most potential index to evaluate individual walking “ability,” however, it is necessary to obtain the ES using 5–8 sets of metabolic measurements at various speeds [4–8, 11]. Each set of metabolic measurement requires 4–5 min, so the participants need to walk at least for more than 20 min. This could not be a heavy exercise for healthy young participants; however, it may be somewhat heavy for physically poor people, such as prosthetic pedestrians, patients after surgery, obese people, pregnant women, and elderly people to execute the whole sets of metabolic measurements. We found that Eff_vert_ was not dependent on walking speeds, at least from 0.667 to 2.028 m s^−1^ on the uphill gradient (Fig. 2a), indicating that it can be evaluated at any designated speed. Furthermore, only two sets of metabolic measurements on the level and shallow uphill gradients without kinematic information are required. This could be a significant reduction of participants’ physical strain during metabolic measurement.

It is worth noting that upward Delta-E value was not significantly different from upward Eff_vert_ values at any speed (Fig. 2a). Methodological considerations should be necessary. Previous studies fixed the horizontal walking speed at 1.2–1.3 m s^−1^ and changed treadmill gradient incrementally [23, 24]. Conversely, we fixed the treadmill gradient at ± 5%, and varied the walking speed incrementally. The pendular energy transduction between kinetic energy and gravitational potential energy became maximal at around 1.4 m s^−1^ [28, 29]. The minimum of the U-shaped relationship between CoT and horizontal walking speed occurs at around 1.4 m s^−1^, irrespective of the gradient [4–6]. These previous findings suggest that the grade incremental protocol may underestimate the EE (≒ higher Delta-E). However, Delta-E reflects the inverse of an increasing rate of mechanical power output to energy consumption rate, so it may not be influenced by walking speed. Indeed, upward Delta-E of 35.5 ± 8.8% was in good agreement with the result of a previous study in young adults involving both genders [23]. Given these considerations, our speed incremental protocol would be equivalent to the grade incremental protocol. We need to remind that Delta-E always requires the inverse “slope” of the regression line of MR_vert_ to *P*_mech_ (Eq. 6). That is, a series of metabolic measurements are necessary to obtain the “slope” from which it is calculated, so it is practically available only for fit populations, but not for clinical patients.

A trend for significantly higher downward Eff_vert_ values at slower *v*_vert_ than at faster *v*_vert_ (Fig. 2b and Additional file 2: Table S1) was consistent with a previous result [25], indicating that the amount of active muscles needed to generate the accelerating force could not be proportional to *v*_vert_ during downhill walking. Indeed, “negative” work rather than “positive” work becomes dominant during downhill walking [25, 26]. The recovery rate of pendular energy transduction gradually decreased at faster *v*_vert_ [26]. This individual variation of the recovery rate of the pendular energy transduction is associated with the muscular EE during negative and positive work. Indeed, the EE of negative work is one third of that of “positive” work [30]. Stored elastic energy in the Achilles tendon and *gastrocnemius medialis* can be utilized even during level walking at 0.75 m s^−1^ [20]. These interactions would be expected to vary considerably between individuals during downhill walking, given that relatively greater variations were observed in downward Eff_vert_ values than in upward Eff_vert_ values (Fig. 2b).

## Conclusions

It is possible to evaluate vertical component of walking efficiency without kinematic information. Upward Eff_vert_ values were nearly constant across a wide range of *v*_vert_, suggesting that EE to lift the body could be proportional to *v*_vert_. Therefore, upward Eff_vert_ should be useful for people with poor physical fitness to evaluate their gait characteristics. This is because only two metabolic measurements are required to obtain individual upward Eff_vert_ on the level and uphill gradients at any voluntary speed. However, this interpretation could not be applied to downhill walking. Delta-E was compatible with upward Eff_vert_, but not with most of the downward Eff_vert_.

## Supplementary information

**Additional file 1: Figure S1.** Relationships between cardiorespiratory responses and walking speed at different gradients.

**Additional file 2: Table S1.** Summary of measured and calculated variables at each speed. P_mech_; mechanical power output for vertical directions, ΔEE; differences of energy expenditure between uphill and level gradients or between level and downhill gradients, MR_vert_; metabolic rate for vertical directions, and Eff_vert_; vertical efficiency. Values are mean (±SD).

## Data Availability

All data used in this study were summarized and in the supplementary information.

## Abbreviations

ANOVA: Analysis of variance
BM: Body mass
CoT: Energy cost of transport per unit distance
Delta-E: Delta efficiency
EE: Whole-body energy expenditure
Eff_vert_: Vertical component of net walking efficiency;
MR_vert_: Vertical component of metabolic rate
*P*_mech_: Vertical component of mechanical power output
VCO_2_: Carbon dioxide output
VO_2_: Oxygen uptake
*v*_vert_: Vertical component of walking speed
ΔEE: Difference of EE between the level and uphill/downhill gradients
