# Supplementary figures and images for: On the simple calculation of walking efficiency without kinematic information for its convenient use
Source: J Physiol Anthropol. 2019 Dec 30;38:17. doi: 10.1186/s40101-019-0211-4 (PMC6937908; doi:10.1186/s40101-019-0211-4)

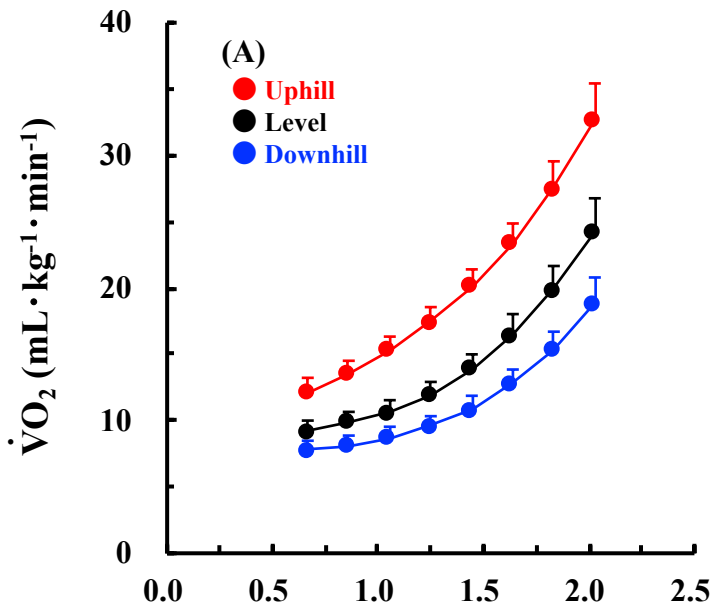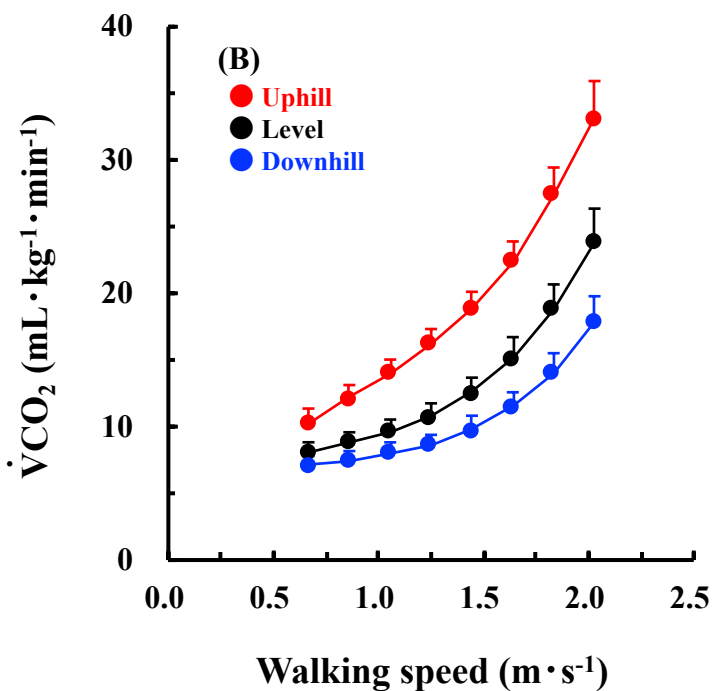

Supplement: Supplementary file 1 — Additional file 1: Figure S1. Relationships between cardiorespiratory responses and walking speed at different gradients. [file 40101_2019_211_MOESM1_ESM.pdf]
