# Supplementary material for: On the simple calculation of walking efficiency without kinematic information for its convenient use
Source: J Physiol Anthropol. 2019 Dec 30;38:17. doi: 10.1186/s40101-019-0211-4 (PMC6937908; doi:10.1186/s40101-019-0211-4)
Supplement: Supplementary file 2 — Additional file 2: Table S1. Summary of measured and calculated variables at each speed. Pmech; mechanical power output for vertical directions, ΔEE; differences of energy expenditure between uphill and level gradients or between level and downhill gradients, MRvert; metabolic rate for vertical directions, and Effvert; vertical efficiency. Values are mean (±SD). [file 40101_2019_211_MOESM2_ESM.pdf]

Supplementary information

Table 1. Summary of measured and calculated variables at each speed.

speed		P_{mech}		ΔEE		MR_{vert}		Eff_{vert}	
horizontal	vertical	Up (n=30)	Down (n=20)	Up	Down	Up	Down	Up	Down
($m \cdot s^{-1}$)		(watt)		($J \cdot kg^{-1} \cdot s^{-1}$)		(watt)		(%)	
0.667	0.033	19.55 (2.21)	19.56 (1.80)	0.938 (0.370)	0.504 (0.194)	56.00 (21.45)	29.86 (10.89)	39.86 (15.34)	74.72 (29.10)
0.861	0.043	25.25 (2.86)	25.26 (2.32)	1.233 (0.278)	0.626 (0.151)	73.31 (16.83)	37.55 (10.13)	35.92 (7.92)	71.67 (18.83)
1.056	0.053	30.95 (3.50)	30.97 (2.85)	1.563 (0.299)	0.745 (0.224)	93.18 (19.76)	44.42 (13.23)	34.40 (7.14)	79.56 (38.26)
1.250	0.063	36.66 (4.15)	36.67 (3.37)	1.865 (0.354)	0.919 (0.226)	111.28 (24.59)	54.83 (13.80)	34.09 (6.76)	71.13 (19.62)
1.444	0.072	42.36 (4.80)	42.38 (3.90)	2.104 (0.427)	1.194 (0.347)	125.92 (31.02)	70.73 (19.75)	35.29 (8.56)	65.43 (24.10)
1.639	0.082	48.06 (5.44)	48.08 (4.42)	2.346 (0.509)	1.413 (0.398)	139.84 (33.76)	84.03 (22.95)	36.04 (8.73)	63.91 (28.36)
1.833	0.092	53.76 (6.09)	53.78 (4.95)	2.628 (0.592)	1.737 (0.334)	156.29 (36.78)	104.46 (24.83)	36.13 (9.01)	53.88 (11.73)
2.028	0.101	59.47 (6.73)	59.49 (5.47)	2.980 (0.567)	2.117 (0.375)	177.74 (38.72)	126.95 (26.80)	34.62 (6.94)	48.45 (8.84)

P_{mech}; mechanical power output for vertical directions, ΔEE; differences of energy expenditure between uphill and level gradients or between level and downhill gradients, MR_{vert}; metabolic rate for vertical directions, and Eff_{vert}; vertical efficiency. Values are mean (±SD).
